# Conjugation of Methotrexate-Amino Derivatives to Macromolecules through Carboxylate Moieties Is Superior Over Conventional Linkage to Amino Residues: Chemical, Cell-Free and *In Vitro* Characterizations

**DOI:** 10.1371/journal.pone.0158352

**Published:** 2016-07-12

**Authors:** Itzik Cooper, Mati Fridkin, Yoram Shechter

**Affiliations:** 1 BBB-group, The Joseph Sagol Neuroscience Center, Sheba Medical Center, Tel Hashomer, Ramat Gan, 52621, Israel; 2 Department of Organic Chemistry, The Weizmann Institute of Science, Rehovot, 76100, Israel; 3 Department of Biomolecular Sciences, The Weizmann Institute of Science, Rehovot, 76100, Israel; National Institutes of Health, UNITED STATES

## Abstract

In this study, we examined the possibility of introducing methotrexate (MTX) to the carboxylate rather than to the ε-amino side chains of proteins. We found that MTX—amino compounds covalently linked to the carboxylate moieties of macromolecules, undergo unusual peptide-bond cleavage, with the release of the MTX amino derivatives from the conjugates. This event takes place at an accelerated rate under acidic conditions, and at a slower rate at physiological pH values. The glutamate portion of MTX is responsible for this behavior, with little or no contribution of the p-aminobenzoate-pteridine ring that is linked to the α-amino side chain of the glutamate. Carboxylate-linked Fmoc-Glu-γ-CONH-(CH_2_)_6_-NH_2_ undergoes hydrolysis in a nearly indistinguishable fashion. A free α carboxylate moiety is essential for this effect. Carboxylate linked Fmoc-glutamic-amide-γ-CONH-(CH_2_)_6_-NH_2_ undergoes no hydrolysis under acidic conditions. Based on these findings, we engineered a cysteine specific MTX containing reagent. Its linkage to bovine serum albumin (BSA) yielded a conjugate with profound antiproliferative efficacy in a MTX-sensitive glioma cell line. In conclusion, carboxylate linked MTX-amino derivatives in particular, and carboxylate linked R-α-GLU-γ amino compounds in general are equipped with‘built-in chemical machinery’ that releases them under mild acidic conditions.

## Introduction

In previous studies we have focused on developing long-acting prodrugs that undergo reactivation in body fluids with desirable pharmacokinetic properties (reviewed in [1, 2]). Our efforts were undertaken predominantly in the fields of diabetes and obesity [3–8]. The “prodrug concept” was introduced in the late 1950’s [9] and is relevant also to the field of cancer therapy, in particular antibody-drug conjugates which have emerged in recent years as promising anti-cancer agents [10]. Low molecular weight anticancer drugs covalently linked to proteins are ordinarily inactive conjugates that can be engineered to undergo reactivation, either enzymatically or chemically upon reaching their target tissues [11].

To date, linkage of low molecular-weight anticancer drugs was conducted through the cysteine moiety or the ε-amino side chains of proteins [12]. In this study we initially linked MTX-amino derivatives to the carboxylate moieties, of a macromolecular model compound, composed of polyethylene glycol of 20-40kDa, containing a single carboxylate, and then to proteins. We report here that such carboxylate-based macromolecule-MTX conjugates have unique, unexpected chemical and pharmacological features. These are described here in detail.

## Materials and Methods

### Materials

Methotrexate, human serum albumin, hexametylenediamine-2HCl, 1.3 diaminopropate-2HCl, 6-aminocaproic-acid, ethylenediamine, dihydrofolate (DHF), N,N dicyclohexylcarbodiimide (DCC), β-nicotinamide adenine dinucleotide 2’ phosphate (NADPH) were purchased from Sigma. Fmoc-Glutamic acid (Fmoc-Glu-OH) and Fmoc-Glutamic acid amide were obtained from DgPeptides Co. LTD (China). PEG_40000_-OSu (mPEG2-N-hydroxysuccinimide ester) was obtained from Shearwater (Huntsville, AL). PEG_20000_-SH (ME-200SH sunbright) and PEG_30000_-SH were purchased from RAPP Polymere (Tuebingen, Germany). All other materials used in this study were of analytical grade.

### Synthesis of MTX-anhydride

Methotrexate (45.4 mg, 100μmoles) was dissolved in 0.9 ml dimethyl sulfoxide (DMSO) and 95 μl from a solution of 1M DCC in DMF (95 μmoles) was then added. The reaction was carried out for 3 hrs at 25°C. Dicyclohexylurea was removed by filtration. The MTX-anhydride formed was kept at 4°C.

### Synthesis of MTX-hexamethylenamine and other MTX-amino derivatives

Hexamethylenediamine-DiHCl (100 μmoles) was dissolved in 1.0ml DMSO, neutralized with N,N-Diisopropylethylamine (DIPEA) and combined with the solution of MTX-anhydride (100 μmoles in 1.0ml DMSO). The reaction mixture was stirred for 4 hrs, precipitated with ethylacetate, washed 4 times with ethylacetate and desiccated. With this procedure, the γ-carboxylate, rather than the α-carboxylate moiety of MTX is predominantly derivatized [13]. MTX-CONH-(CH_2_)_6_-NH_2_ was obtained in 60% yield. It has the molar extinction coefficient similar to MTX (ε_305_ = 22700, ε_372_ = 7200). MTX-CONH-(CH_2_)_2_-NH_2_, MTX-CONH-(CH_2_)_3_-NH_2_ and MTX-CONH-CH-(COOH)-(CH_2_)_4_-NH_2_ were synthesized by the same procedure applied to MTX-hexamethylene-amine except that hexamethylene-diamine was replaced by ethylenediamine, 1.3 diaminopropane-2HCl, or Nε-t-BOC-L-lysine respectively. The protecting group was then removed by trifluoroacetic acid. The calculated molecular weights for MTX-CONH-(CH_2_)_2_-NH_2_ is 496 Da, found by ESMS M+H = 497.36, that for MTX-CONH-(CH_2_)_3_-NH_2_ is 539, found by ESMS M+H = 540.45 and for MTX-CONH-(CH_2_)_6_-NH_2_ is 552 Da, found for M+H = 553.41 Da and for MTX-CONH-CH-(COOH)-(CH_2_)_4_-NH_2_ is 682 Da, found M+H = 683.37 Da.

### Preparation of PEG_40_-CONH-(CH_2_)_6_-NH_2_

Solid PEG_40_-OSu (280mg ~ 7 μmoles) was added to 5.0 ml hexamethylenediamine-2HCl (1M in 0.1M Hepes buffer pH 7.4 precooled to 0°C). The reaction was carried out for 5 hrs at 0°C, with stirring. The derivative obtained was dialyzed against H_2_O with several changes of H_2_O over a period of 3 days and then lyophilized.

### Preparation of PEG_40_-CONH-(CH_2_)_5_-COOH

This derivative was prepared by the same procedure applied for PEG_40_-CONH-(CH_2_)_5_-NH_2_ except that hexamethylenediamine was replaced by 6-aminocaproic acid. The product thus obtained was extensively dialyzed against H_2_O and then lyophilized.

### Synthesis of cysteine-specific-MTX-containing reagents [MAL-(CH_2_)_2_-CONH-(CH_2_)_2,3,6_-NHCO-MTX]

Solutions of MTX-CONH-(CH_2_)_2_-NH_2_, MTX-CONH-(CH_2_)_3_-NH_2_ or MTX-CONH-(CH_2_)_6_-NH_2_ (100 μmoles of each in 1 ml DMSO) were neutralized with one equivalent of DIPEA (100μmoles, 17.4 μl), and each combined with 120μmoles of MAL-(CH_2_)_2_-COOSu (32mg in 0.3ml DMSO). The reaction was carried out for 3 hrs at 25°C with stirring. The products were precipitated with ethylacetate, washed 5 times with this solvent, twice with ethylether and dried in Vacuo. The products obtained were characterized for their calculated molecular weights by mass spectroscopy and for having a 1:1 MTX to maleimide molar ratio. MTX was quantitated by its absorbance at 372 nm (ε_372_ = 7200) and the alkylating capacity of MAL was determined by reacting the reagents with reduced glutathione (GSH) and determining the amount of GSH consumed with 5,5’-dithio-2 nitrobenzoic acid (DTNB). No significant decrease in the alkylating capacity of the maleimido group was detected during storage of those compounds, in their solid state, over a period of several months.

### Derivatization of BSA and HSA with the cysteine-specific MTX-containing reagents

70mg (1 μmole) of HSA and BSA were dissolved on 2.0 ml of 0.05M Hepes buffer (pH 7.3). this was followed by the addition of cysteine specific reagents (i.e. MAL-(CH_2_)_2_-CONH-(CH_2_)_2,3,6_-NHCO-MTX, 3μmoles, 30μl from a solution of 100μmoles/ml in DMSO). The reaction was carried out for 1 hr at 0°C with stirring. The protein derivatives (20mg of each) were loaded on Sephadex-G-50 columns (14x1.5cm) that were equilibrated and run with 0.01M NaHCO_3_. The fraction corresponding to the protein derivatives were collected and lyophilized. This procedure removed unreacted as well as non-covalently adsorbed MTX derivatives to the protein.

### Preparation of PEG_40_-CONH-(CH_2_)_6_-NHCO-MTX (compound A, Table 1)

**Table 1:** Principle macromolecules conjugates, prepared and investigated in this study.

Conjugate number	Abbreviated structure
**A**	PEG_40_-CONH-(CH_2_)_6_-NH**CO-MTX**
**B**	PEG_40_-CONH-(CH_2_)_5_-CO**NH-(CH**_**2**_**)**_**6**_**-NHCO-MTX**
**C**	PEG_20_-S-MAL-(CH_2_)_2_-CO**NH-(CH**_**2**_**)**_**6**_**- NHCO-MTX**
**D**	HSA-S-MAL-(CH_2_)_2_-CO**NH-(CH**_**2**_**)**_**6**_**- NHCO-MTX**
**E**	HSA-S-MAL-(CH_2_)_2_-CO**NH-(CH**_**2**_**)**_**2**_**- NHCO-MTX**
**F**	HSA-S-MAL-(CH_2_)_2_-CO**NH-(CH**_**2**_**)**_**3**_**- NHCO-MTX**
**G**	HSA-S-MAL-(CH_2_)_2_-CO**NH-CH-(COOH)-(CH**_**2**_**)**_**4**_**- NHCO-MTX**
**H**	PEG_30_-S-MAL-(CH_2_)_2_-CO**NH-(CH**_**2**_**)**_**6**_**- NH-Glu-Fmoc**
**I**	PEG_30_-CONH-(CH_2_)_6_-**NHCO-Glu-Fmoc**
**J**	PEG_30_-S-MAL-(CH_2_)_2_-CO**NH-(CH**_**2**_**)**_**6**_**- NH-Glu-amide-Fmoc**
**K**	BSA-S-MAL-(CH_2_)_2_-CO**NH-(CH**_**2**_**)**_**6**_**- NHCO-MTX**
**L**	BSA---NH**-CO-MTX**

PEG_40_-CONH-(CH_2_)_6_-NH_2_ (40 mg ~ 1 μmole) dissolved in 1.0 ml 0.1M Hepes buffer pH 7.4, cooled to 0°C, and 7 aliquots of 10 μl MTX-anhydride each (~1 μmole) from a solution of 100 μmoles/ml in DMSO were added over a period of 40 min. The reaction mixture was incubated for an additional 2 hrs and then dialyzed against H_2_O with several changes of H_2_O over a period of 2 days and lyophilized.

### Preparation of PEG_40_-CONH-(CH_2_)_5_-CONH-(CH_2_)_6_-NHCO-MTX (compound B, Table 1)

PEG_40_-CONH-(CH_2_)_5_-COOH (40 mg ~ 1μmole) was dissolved in 2ml DMSO. N-hydroxysuccinimide (~7 μmoles) and DCC (1 μmole) was then added. The reaction was carried out for 1 hr at 25°C with stirring. MTX-hexamethyleneamine (30 μl from a solution of 100 μmoles/ml in DMSO, ~3μmoles) was then added. The reaction was carried out for an additional two hrs. The conjugate thus obtained was precipitated with ether, washed three times with ether and desiccated. The product was then dissolved in 1.0 ml 0.01M NaHCO_3_ (pH ~ 7.4), dialyzed for 10 hrs against the same buffer at 4°C, and lyophilized.

### Synthesis of Fmoc-L-glutamic acid anhydride

Fmoc-L-glutamic acid (369mg, 100μmoles) dissolved in 0.9 ml DMSO, and 95μl from a solution of 1M DCC in DMF (95 μmoles) was then added. The reaction was carried out for 3 hrs at 25°C with stirring. Dicyclohexylurea was removed by filtration. The product formed was kept at 4°C until used.

### Synthesis of Fmoc-glutamic acid-hexamethyleneamine

Hexamethylenediamine-DiHCl, (100μmoles) was dissolved in 1.0 ml DMSO, neutralized with DIPEA, and combined with a solution of Fmoc-glutamic acid anhydride (100 μmoles). The reaction mixture was stirred for 2 hrs, precipitated with ether, washed 3 times with ether, twice with ethylacetate and desiccated. Fmoc-L-Glu-CONH-(CH_2_)_6_-NH_2_ was obtained in 74% yield. It has a molar extinction coefficient of ε_280_ = 7200±100. The calculated molecular weight is 467 Da, found by ESMS 468.29 for M+H and 490.38 for M+Na.

### Synthesis of Fmoc-L-glutamic acid amide-γ-hexamethyleneamine

F-moc-glutamic-amide (37mg, 100μmoles) was dissolved in 0.1ml DMSO and mixed with 100μl of 110μmoles hexamethylene-diamine 2HCl, that was neutralized with DIPEA. DCC, 0.1 ml from a solution of 1M in DMF (100μmoles) was then added. The reaction was carried out for 4 hrs at 25°C. DCU was removed by precipitation and the product obtained was precipitated with ether, washed 3 times and desiccated. The product was obtained in 64% yield. It has a molar extinction coefficient of ε_280_ = 7200±100. The calculated molecular weight is 466 Da, found by ESMS for M+H 467.29.

### Preparation of PEG_30_-S-MAL-(CH_2_)_2_-CONH-(CH_2_)_6_-NH-Glu-Fmoc (compound H, Table 1)

Fmoc-Glu-hexamethyleneamine (2.81 mg, ~6 μmoles) was dissolved in 50 μl DMSO and combined with (1.07 mg, ~4μmoles) MAL-(CH_2_)_2_COOSu, also dissolved in 50μl DMSO. The reaction was carried out for 3 hrs with stirring and then added to a solution of PEG_30_-SH (100 mg ~3.3 μmoles) in 0.02 M Hepes buffer (pH 7.4) precooled to 0°C. The reaction was carried out for 30 min and then dialyzed overnight against 0.01M NaHCO_3_ at 4°C with two changes of this buffer and lyophilized. The conjugate obtained contains 0.8±0.1 mole Fmoc-Glu per mole (40 mg solid material) as judged by its absorption at 280nm (ε_280_ = 7200) and by its glutamic acid content following acid hydrolysis and quantitative amino acid analysis.

### Preparation of PEG_40_-CONH-(CH_2_)_6_-NHCO-Glu-Fmoc (compound I, Table 1)

PEG_40_-CONH-(CH_2_)_6_-NH_2_ (80mg, ~2 μmoles) was dissolved in 2.0 ml of 0.02M Hepes buffer (pH 7.4), cooled to 0°C and then 6 aliquots of Fmoc-glutamic-acid-anhydride (20 μl each, ~2 μmoles) from a solution of 100 μmole/ml in DMSO were added over a period of 1 hr. The reaction mixture was incubated for an additional 2 hrs at 0°C, and then dialyzed against H_2_O with several changes of H_2_O over a period of 2 days and lyophilized. The product thus obtained, contains 0.7 mole Fmoc-glu per mole conjugate (40 mg solid material) as judged by its absorbance at 280 nm (ε_280_ = 7200) and by its content of glutamic acid following acid hydrolysis and quantitative amino-acid analysis.

### Preparation of PEG_30_-S-MAL-(CH_2_)_2_-CONH-(CH_2_)_6_-NH-Glutamic acid-amide-Fmoc (compound J, Table 1)

This derivative was prepared by the same procedure applied for compound H, (Table 1) except that F-moc-Glu-hexametheyleneamine was replaced with Fmoc-Glu-amide-hexamethyleneamine.

### Partial purification of dihydrofolate reductase (DHFR) from chicken liver

This was carried out by the procedure of Kaufman and Kemerer [14] with some modifications: Fresh chicken liver (20 grams) was cut into pieces, homogenized in 0.1M MnCl_2_ and centrifuged. The supernatant was brought to pH 5–6 with HCl, and ZnSO_4_ was added gradually to obtain a final concentration of 20 mM. The pH was elevated to pH 7.5–8 by the addition of solid NaHCO_3_. The precipitate formed (about 87% of total protein) was removed by centrifugation. The proteins remaining in the supernatant were precipitated with 4.54 M ammonium sulfate. The precipitate was then dissolved in 50 mM Tris-HCl (pH 7.5) containing 0.1M KCl, dialyzed for two days against the same buffer and then frozen in aliquots at -80°C until used. Partially purified DHFR prepared by this procedure has a specific activity of about 0.05 units/mg.

### Cell-free enzymatic assay for DHFR

The assay was based on DHFR dependent reduction of DHF to tetrahydrofolate. In this process NADPH is oxidized to NADP, and the extent of the oxidation is monitored by the decrease in absorbance at 340 nm. Tubes containing 1.0 ml each of 0.05M Tris-HCl buffer (pH 7.5), 0.5M KCl, 0.137 mM NADPH and 0.187 mM DHF were incubated for 40 min at 25°C in the absence and the presence of partially purified DHFR (35μg/ml), and increasing concentrations of MTX or its derivatives. The absorbance at 340 nm was then monitored. In a typical assay, absorbance at 340 nm amounted to 1.7±0.06 and 0.65±0.03 in the absence and the presence of DHFR. MTX inhibits DHFR with an IC_50_ value of 0.08 μM (80 picomoles/ml). A methotrexate derivative yielding an IC_50_ value of 0.8 μM was considered as having 10% the inhibitory potency of the native folic acid antagonist.

### Growth inhibition effects of BSA-MTX analogues

The MTX-sensitive glioma cell line CNS-1 was grown in 96 well plates in DMEM containing 10% fetal calf serum; 2mM L-glutamine, penicillin (100 units/ml) and streptomycin (0.1 mg/ml) under humidified atmosphere containing 5% CO_2_. Cells were seeded at 1,000cells/well. 24hrs later, MTX or BSA-MTX analogues were added to each plate to give a final MTX or albumin-MTX analogue concentration as indicated in the text. Cell viability was measured after 72 h using a standard MTT (3-(4,5-dimethylthiazol-2-yl)-2,5-diphenyltetrazolium bromide) assay as described before [15]. Experiments were repeated at least 3 times in quadruplicate. IC_50_ values were calculated from dose response curves using a median-effect plot.

### Statistical analysis

Statistical analyses were performed using the Prism 4 software. Data are presented as the means ± standard error of the mean (SEM) or standard deviation (STDEV). Differences between two groups were assessed by an unpaired t-test and among three or more groups by a one-way analysis of variance followed by Tukey’s Multiple Comparison Test. A p-value of less than 0.05 was considered to be statistically significant.

## Results

### Covalently-linked MTX-hexamethylenamine to PEG_40_-------COOH, undergoes cleavage under acidic conditions

**Fig 1:**
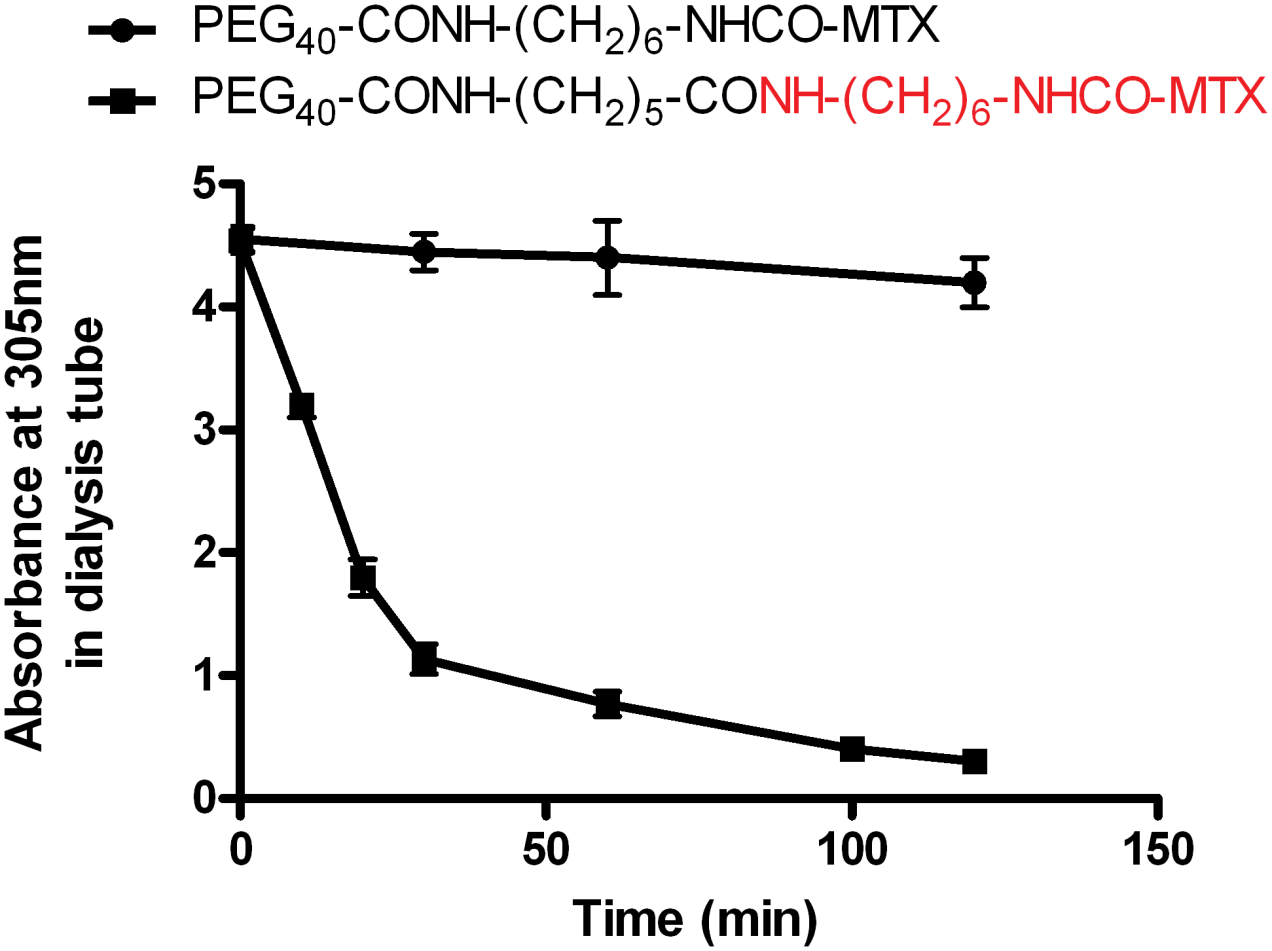
Decrease in the absorbance of PEG_40_-CONH-(CH_2_)_5_-CONH-(CH_2_)_6_-NHCO-MTX with time during dialysis against 1mM HCl. PEG_40_-CONH-(CH_2_)_5_-CONH-(CH_2_)_6_-NHCO-MTX and PEG_40_-CONH-(CH_2_)_6_-NHCO-MTX (0.2 μmole/ml of each) were dialyzed against one liter of 1mM HCl (pH 3.0). At the indicated time points, 30 μl aliquots were withdrawn from the dialysis tube, diluted 20 folds and their absorbance at 305 nm was monitored. Data are presented as mean±STDEV (n = 3).

**Fig 2:**
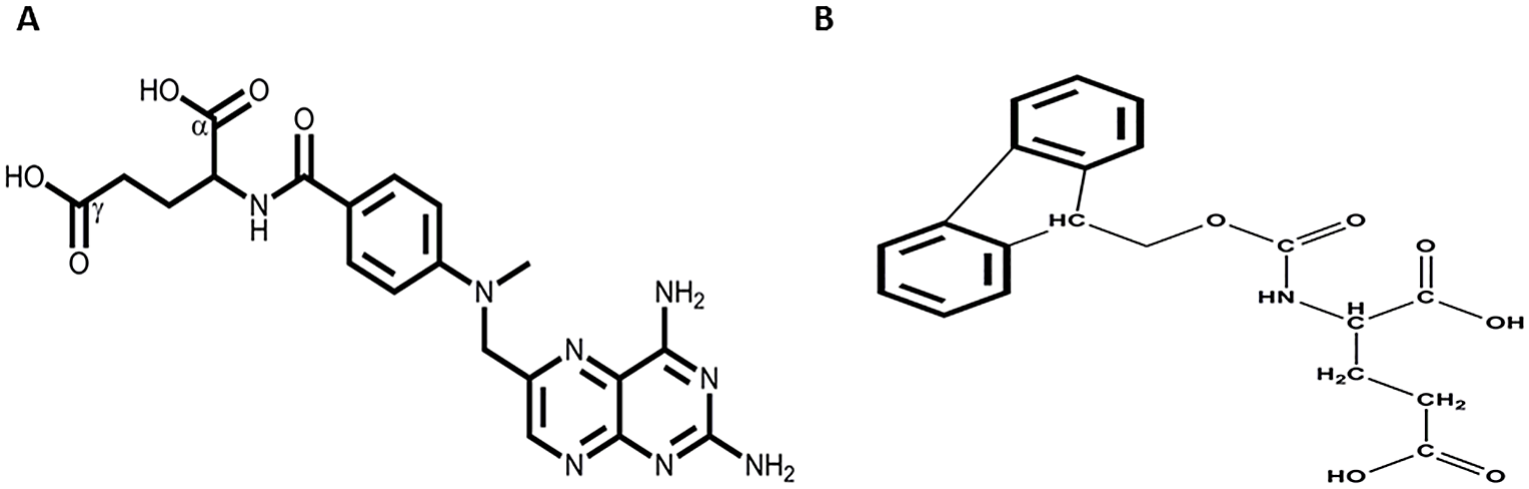
MTX and Fmoc-L-glutamic acid structures. Illustrations of MTX (A) and Fmoc-L-glutamic acid (B).

In Table 1 we have listed the abbreviated structures of all macromolecular conjugates prepared and studied. In the first two, MTX was linked either to an amino side-chain (conjugate A) or a carboxylate moiety (conjugate B), located on a polyethylene glycol chain of 40kDa. The two conjugates (0.2μmol/ml) were dialyzed against one liter of 1 mM HCl (pH 3.0) and the absorbance at 305nm in the dialysis tubes was recorded as a function of time. In contrast to the amino-linked PEG_40_-MTX analogue (conjugate A), in conjugate B the absorbance at 305nm was substantially decreased within minutes and about 80% was dialyzed within a period of two hours (Fig 1). This finding suggested to us that the peptide bond connecting MTX-hexamethylenamine to this carboxylate-containing macromolecule, undergoes cleavage under acidic conditions. Fig 2 illustrates the structures of MTX and Fmoc-L-glutamic acid used in this study.

### Characterizing the released MTX-hexamethylenamine

**Fig 3:**
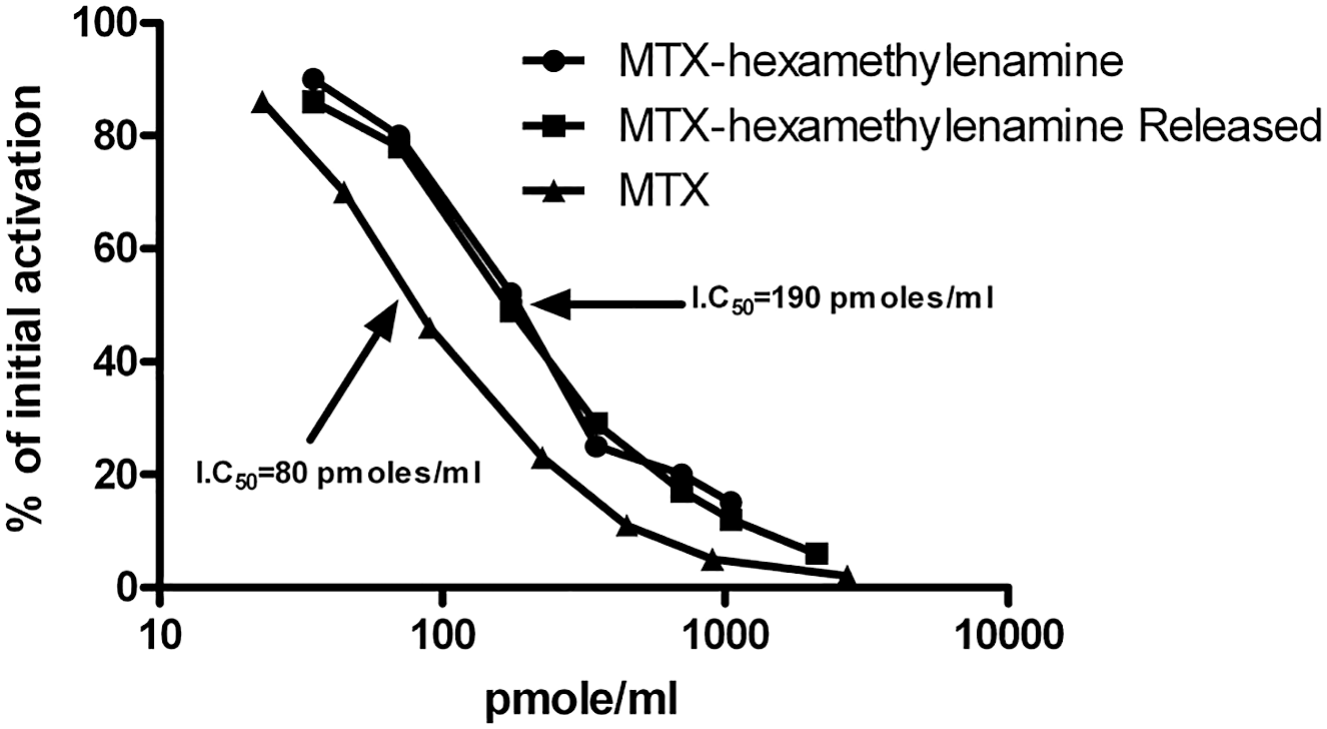
Dose response inhibition of dihydrofolate-reductase by MTX-hexamethylenamine, released from PEG_40_-CONH-(CH_2_)_5_-CONH-(CH_2_)_6_-NHCO-MTX at acidic conditions. The indicated concentrations of MTX and its derivatives were analyzed for their potencies to inhibit the reduction of dihydrofolate to tetrahydrofolate by DHFR. In this process NADPH is oxidized to NADP, and the extent of this oxidation is monitored by the decrease in absorbance at 340 nm (experimental section).

To verify this point, the conjugate (2μmoles in 1ml) was dialyzed against 15 ml of 1.0 mM HCl over a period of 24 hrs. The dialyzed fraction was collected, lyophilized and characterized. Table 2 summarizes several features of the collected fraction. It absorbs at 305 and 372 nm with similar extinction coefficients characterizing MTX or MTX-amino derivatives. It has 1.0±0.1 mol/mol glutamic acid, the corrected mass of MTX-hexamethylenamine (552.3±0.04 Da) and it inhibits DHFR with equipotent efficacy to that of MTX-hexamethylenamine (IC_50_ = 190±7 picomoles/ml, Table 2, Fig 3).

**Table 2:** Features of MTX-hexamethylamine released from PEG_40_---------CONH-(CH_2_)_6_-NHCO-(CH_2_)_6_-NH-CO-MTX at acidic conditions^a^.

Characteristic	
**Absorbance at 372 nm**	ε_305_ = 7200
**Absorbance at 305 nm**	ε_305_ = 22.700±100
**305/372 ratio**	3.152
**Glutamic acid content**^b^	1.0±0.1 mol/mol
**Mass spectrum**^c^	Calculated 552 Da, found ES+552.30±0.04Da
**Inhibition of DHFR**^d^	IC_50_ = 190±7 pmoles/ml

^(a)^ PEG_40_-CONH-(CH_2_)_6_-CONH-(CH_2_)_6_-NHCO-MTX (2 μmol/1.0 ml) was dialyzed against 15 ml of 1 mM HCl, for 24 hrs. The fraction dialyzed-out was collected, lyophilized, dissolved in 1.0 ml H_2_O and subjected for further characterization.

^(b)^ Derivative concentration was determined by its absorbance at 372 nm, a 20μl aliquot was acid hydrolized and glutamic acid quantitated by amino-acid analysis.

^(c)^ Mass spectrum was determined by the electrospray ionization technique.

^(d)^ Assay is based on DHFR dependent reduction of dihydrofolate to tetrahydrofolate.

### Rates of hydrolysis of PEG_40_-----------CONH-(CH_2_)_6_-NHCO-MTX at acidic and neutral pH values

**Fig 4:**
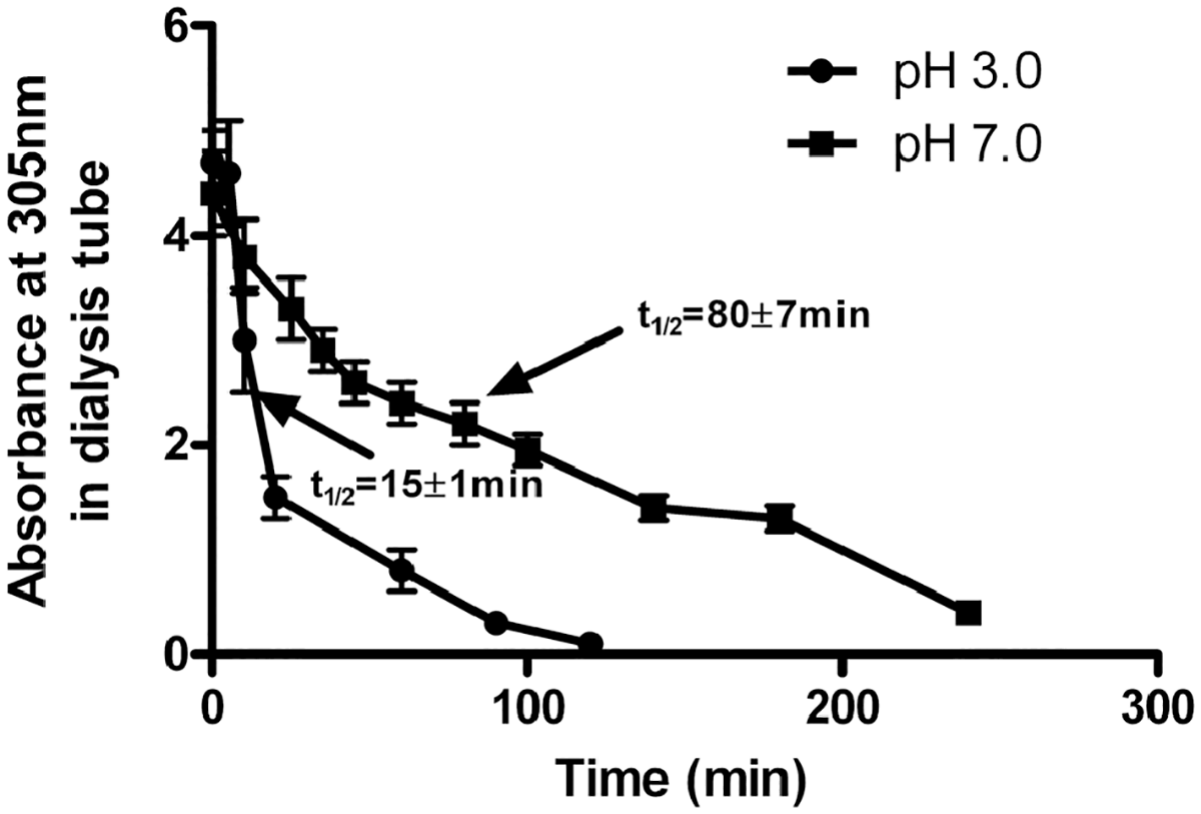
Rate of hydrolysis of MTX-CONH-(CH_2_)_6_-NH_2_ from PEG_40_-CONH-(CH_2_)_5_-CONH-(CH_2_)_6_-NHCO-MTX at acidic and neutral pH values. Rate of hydrolysis was determined by the decrease in the absorbance of the PEG_40_-MTX conjugate at 305nm as a function of time during dialysis against 1mM HCl (pH 3.0) or 50 mM Hepes buffer (pH 7.4). The experimental protocol described in the experimental part was applied. Data are presented as mean±STDEV (n = 4).

Fig 4 shows the rate of hydrolysis of this conjugate using the experimental protocol applied in Fig 1. The conjugate lost its absorbance in the visible region (at 305nm) when dialyzed at pH 3.0 with a t_1/2_ value of 15±1 min, while at pH 7.4, t_1/2_ value was 80±7 min (Fig 4). Thus, the rate of hydrolysis of such carboxylate-based conjugate is strongly acid-pH dependent. It exceeds by 5.3 fold the rate obtained at physiological pH.

### Designing a cysteine-specific-MTX-containing reagent that releases the MTX-amino compound under acidic conditions

**Fig 5:**
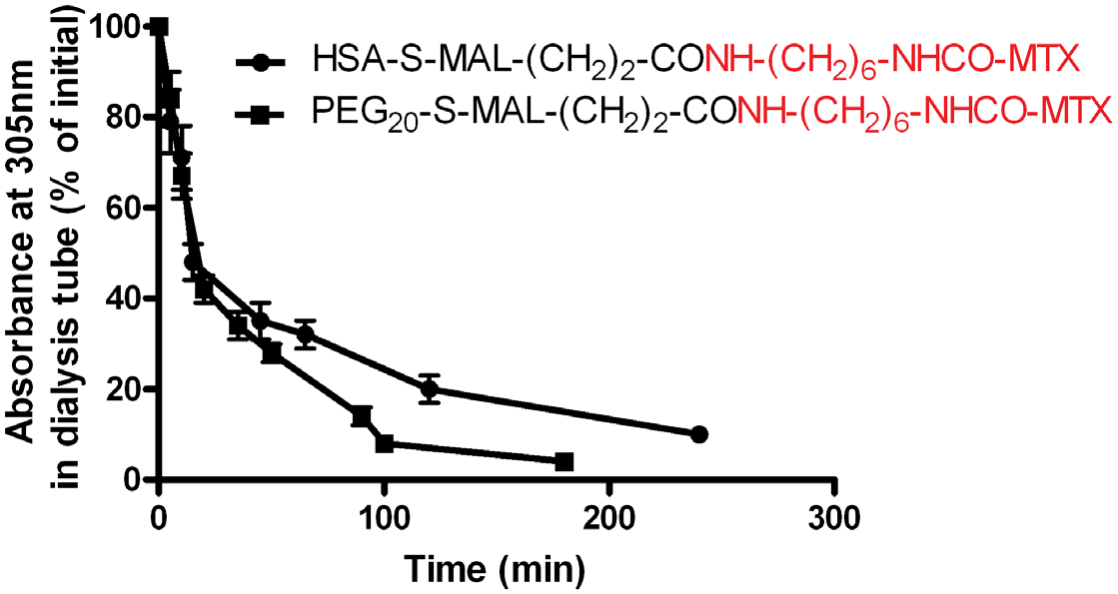
Engineering a cysteine-specific MTX-containing reagent- Rates of hydrolysis following its conjugation to PEG_20_-SH or to HSA at acidic pH. PEG_20_-S-MAL-(CH_2_)_2_-CONH(CH_2_)_6_-NHCO-MTX and HSA-S-MAL-(CH_2_)_2_-CONH(CH_2_)_6_-NHCO-MTX were dialyzed against 1L of 1mM HCl and the decrease in the absorbance at 305nm in the dialysis tube was recorded applying the protocol as described in the legend for Fig 1. Data are presented as mean±STDEV (n = 4).

Based on the above findings, we hypothesized that a cysteine-specific reagent consisting of maleimide (MAL) on one hand, MTX on the other, and the type of peptide bond which undergoes MTX-dependent hydrolysis, can be engineered. Such a reagent was prepared, linked to PEG_20_-SH (conjugate C, Table 1) and analyzed for its rate of hydrolysis at pH 3.0. As found for conjugate B, it was hydrolyzed with a t_1/2_ value of 17±2 min and in a nearly quantitative fashion within a period of 2 hrs (Fig 5). We subsequently linked this reagent to the single cysteinyl moiety of HSA (conjugate D, Table 1). This conjugate was hydrolyzed as well at pH 3.0 (Fig 5) and at pH 7.4 (not shown) in a nearly indistinguishable manner to that obtained with conjugate B.

### Structural alterations in the cysteine-specific MTX-containing reagent: Effect on the rates of hydrolysis

In order to obtain more structural data on this acid dependent chemical hydrolysis, we compared the hydrolysis rates of conjugates D, E, F, and G (Table 1) at pH 3.0. Those differ from each other in the type of the spacer arm connecting MTX to the cleavable peptide bond (Table 3). Conjugate E, having the shorter version of spacer arm (two ethylene groups), hydrolyzed at the fastest rate (t_1/2_ = 6±0.7 min) exceeding in rate nearly 3 times that of conjugate D (six ethylene groups, t_1/2_ = 17±2 min, Table 3). Conjugate F (3 ethylene groups) hydrolyzed at the slowest rate (t_1/2_ = 58±3 min, Table 3). The lysine containing analogue G hydrolyzed at a fast rate as well. In this analogue, the spacer arm differs from the others in containing additional carboxylate moiety, one bond away from the cleavable peptide bond (Tables 1 and 3).

**Table 3:** T_1/2_ values for the rates of hydrolysis of HSA-S-MAL----CO~~MTX analogues at pH 3.0: Dependency on the spacer-arm connecting MTX to the cleavable peptide bond.

Conjugate number	Spacer arm	Hydrolysis rates (pH 3.0) T_1/2_ values (min)
**D**	H_2_N-(CH_2_)_6_-NH_2_	17±2^1^
**E**	H_2_N-(CH_2_)_2_-NH_2_	6±0.7
**F**	H_2_N-(CH_2_)_3_-NH_2_	58±3
**G**	H_2_N-CH-COOH-(CH_2_)_4_-NH_2_	7±0.7

^(1)^Values derived from Fig 5

### Role of the non-glutamate portion of MTX and the contribution of its α-carboxylate moiety to the acid-dependent hydrolysis

**Fig 6:**
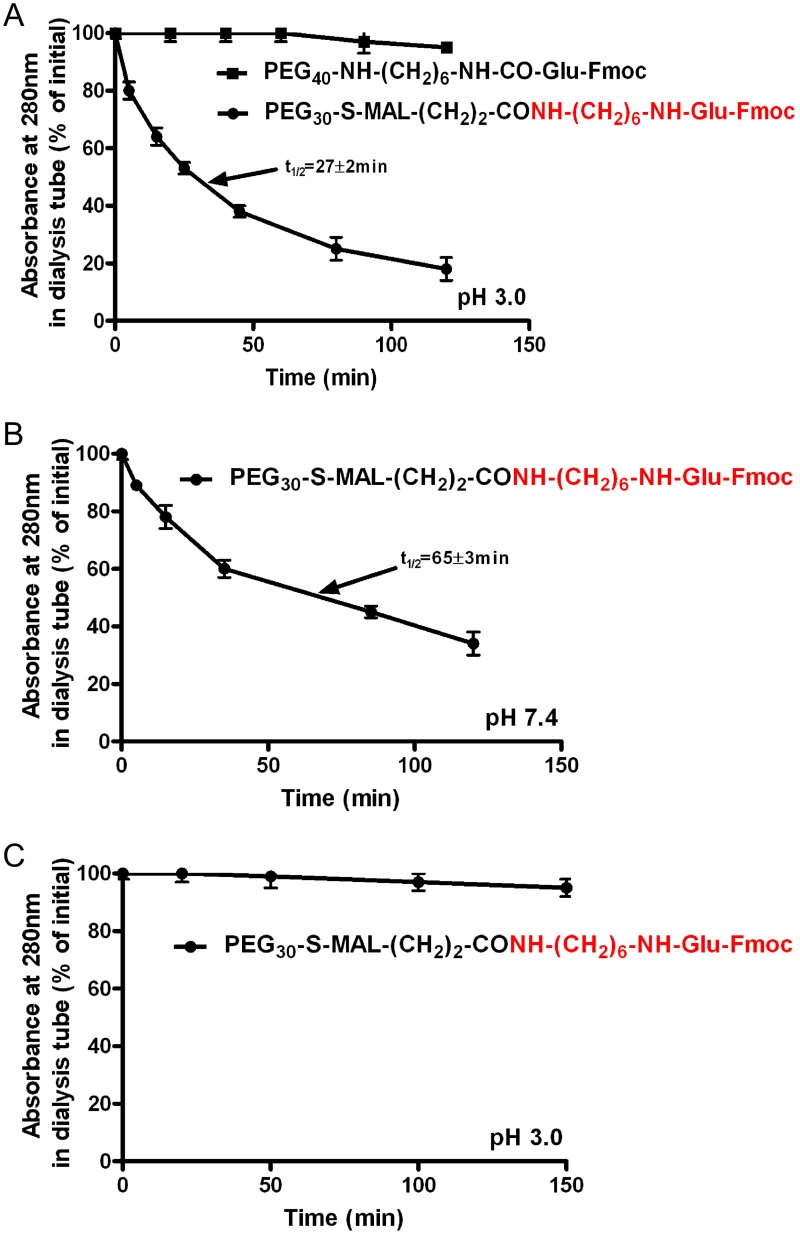
Rate of hydrolysis of F-moc-L-glutamic-hexamethylenamine linked to PEG_30_-----COOH at acidic and neutral pH values. The rates of hydrolysis were determined by the decrease in absorbance of the conjugates at 280nm as a function of time during dialysis against 1mM HCL (pH 3.0, Fig 5A and 5C) or PBS (pH 7.4, Fig 6B). Data are presented as mean±STDEV (n = 4).

MTX is composed of L-glutamic acid linked through its α-amino side chain to p-aminobenzoate-pteridine ring (Table 1). In order to determine whether this non-glutamate portion of MTX is required for the observed acid-dependent hydrolysis, we have prepared PEG_30_**--------**COOH linked to Fmoc-L-glutamate-CONH-(CH_2_)_6_-NH_2_ (compound H, Table 1). As shown in Fig 5A and 5B, this analogue was hydrolyzed both at pH 3.0 (t_1/2_ = 27±2 min) and at pH = 7.4 (t_1/2_ = 65±3 min) in a nearly indistinguishable pattern to that obtained with the equivalent MTX-containing analogues (Figs 3 and 4). Thus, the non-glutamate portion of MTX has no role in facilitating hydrolysis and essentially can be replaced by any other R-group-located at the N-terminal side chain of glutamic acid. Finally, we have prepared PEG_30_ **------**COOH linked to Fmoc-glutamic acid-amide, an analogue in which the α-carboxylate moiety of glutamic acid is amidated (compound J, Table 1) and found that this analogue undergoes no hydrolysis under acidic conditions (Fig 6C). Thus, the free, non-derivatized α-carboxylate moiety of glutamate is essential for this chemical based hydrolysis.

### Enhanced antiproliferative efficacy by hydrolyzable BSA-MTX conjugate in a CNS-1 glioma cell line

**Fig 7:**
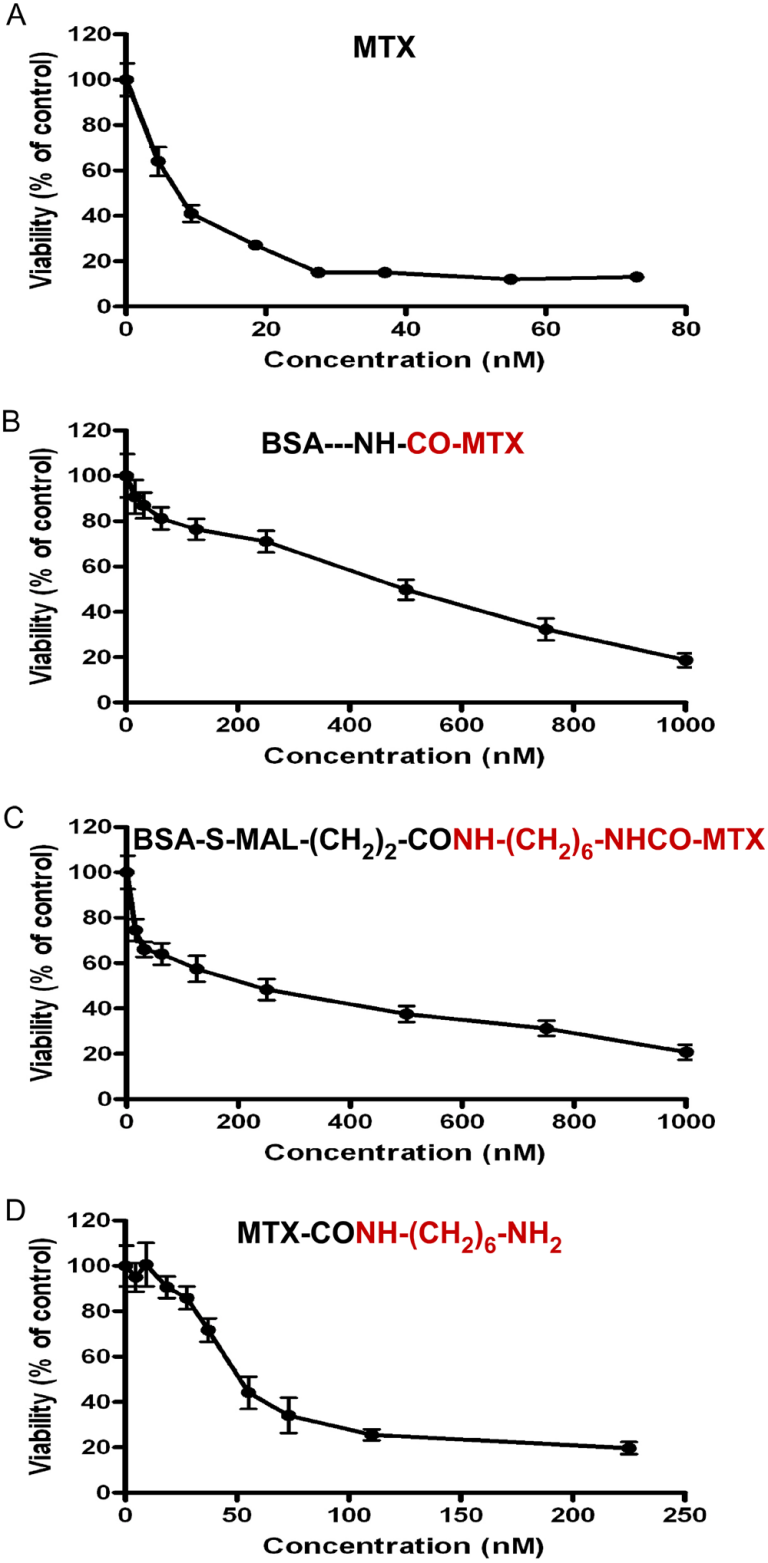
Enhanced antiproliferative efficacy by hydrolysable BSA-MTX conjugate is CNS-1 glioma cell line. Dose dependent toxicity experiments were conducted on CNS-1 cells as described in the Methods section. MTX (A), BSA---NH-CO-MTX (B) and BSA-S-MAL-(CH_2_)_2_-CONH-(CH_2_)_6_- NHCOMTX (C) were added to the cell culture for 72 hrs before MTT toxicity assay was applied to determine their antiproliferative efficacies. Data are presented as mean±SEM of 3–4 experiments in quadruplicates.

Fig 7 shows the dose-dependent killing potency of a BSA version of our cysteine-specific MTX-containing analogue (conjugate K in Table 1). This was compared to an analogue in which MTX was covalently linked to the lysine moieties of BSA (conjugate L, Table 1). This study was carried out in a rat-gioma cell line selected to be highly sensitive to the modulating action of MTX (Fig 7A). Both analogues are antiproliferatively active, however conjugate K shows IC_50_ value of 140±17 nM, as opposed to an IC_50_ value of 381±56 nm found for conjugate L (Fig 6B and 6C and summarized in Table 4). Non-covalently linked MTX-CONH-(CH_2_)_6_-NH_2_ facilitates its antiproliferative effect with an IC_50_ value of 67±4nM (Fig 7D, Table 4), indicating that conjugate K (IC_50_ = 140nM) preserves a substantial amount (48%) of its antiproliferative efficacy following the covalent introduction of the chemically-releasable MTX-amino compound to this carrier protein.

**Table 4:** An antiproliferative efficacy of BSA-MTX conjugates against MTX-sensitive CNS-1 cells.

Compound	IC_50_ (nM)
**Methotrexate**	6.8±0.8
**BSA-S-MAL-(CH**_**2**_**)**_**2**_**-CONH-(CH**_**2**_**)**_**6**_**- NHCO-MTX**^(1)^	140±17**
**BSA---NH-CO-MTX**^(2)^	381±56
**MTX-CONH-(CH**_**2**_**)**_**6**_**-NH**_**2**_	67±9

^(1)^Conjugate K in Table 1.

^(2)^Conjugate L in Table 1.

** p<0.01 vs. BSA---NH**-CO-MTX** (student t-test).

## Discussion

In general, the covalent conjugation of low molecular-weight drugs to proteins yields inactive products [16, 17]. This phenomenon has profound clinical advantages if the conjugate releases the drug in its active form upon reaching its target tissues [18]. With regard to protein-methotrexate conjugates, this folate antagonist was linked through a variety types of linkers, either to a cysteinyl moiety or in cysteinyl-deficient proteins, to the ε-amino side chains of lysines [19, 20]. Reactivation has been achieved, either by intracellular (lysozomal) proteolysis of the conjugate, yielding short MTX-containing active fragments, or by including a cleavable site in the spacer connecting MTX to the protein to permit cleavage by proteases present at the sites of inflammation [21].

In this study, we examined whether the covalent introduction of MTX, to the carboxylate moieties, rather than to the ε-amino side chains of proteins would be of value and offer potential clinical benefit. As a model for macromolecule-MTX conjugates, we first prepared polyethylene-glycol chains of 40 kDa containing either a single amino side chain or a carboxylate moiety. Methotrexate was covalently linked to each of those to obtain either amino-linked or carboxylate linked PEG_40_-MTX conjugates having the abbreviated structures summarized in Table 1.

Surprisingly, overnight dialysis of conjugate B (Table 1) against 1 mM HCl (pH 3.0) led to a total loss in the absorbance of this conjugate in the visible region (i.e. at 305 and 372 nm). This was found to be unique to this analogue, and was not shared with the amino-linked PEG_40_-MTX conjugate (Fig 1). We hypothesized that conjugate B (Table 1) uniquely undergoes acid-based chemical catalysis with the concomitant hydrolysis of the peptide-bond connecting PEG_40_---------COOH to MTX-hexamethylenamine. This point was subsequently verified. The dialyzed fraction was collected and found to be identical to free MTX-hexamethylenamine by four different criteria, including an equipotent dose-dependent efficacy of DHFR inhibition (Fig 3, summarized in Table 2).

Based on these findings, we subsequently designed a cysteine-specific-MTX-containing reagent. It contains maleimide on one side, the MTX on the other side, and the peptide bond in the correct orientation to generate MTX-dependent-acidic dependent peptide bond cleavage. Its linkage to PEG_20_-SH or to the single-cysteinyl moiety of HSA, yielded conjugates that undergo acid-dependent hydrolysis in a similar quantitative and qualitative fashion, to that obtained with conjugate B (Table 1) respectively (Fig 4 and Table 3). Thus, the high reactivity of maleimide toward sulfhydryl moieties [22] enabled generation of specifically labeled conjugates that release MTX-amino compounds under acidic conditions.

We next aimed to gain mechanistic insight into this peculiar chemical hydrolysis. For that reason, we first prepared a repertoire of HSA-MTX derivatives which differ in the length of the diamino spacer-arm bridging between MTX and the cleavable peptide bond. All were found to facilitate hydrolysis, some at a more accelerated rate (i.e. spacer arm H_2_N-(CH_2_)_2_-NH_2_) or at a slower rate (i.e. spacer arm H_2_N-(CH_2_)_3_-NH_2_, Table 3). We next found that the non L-glutamic acid portion of MTX is not required to manifest this chemical hydrolysis. The P-aminobenzoate-pteridine ring can be replaced by other R-groups (Fmoc in this study) covalently linked to the α-amino side chain of glutamic acid (Fig 6). This observation extends this finding to other drug delivery systems as well as toward developing new versions for protracting the life-time of peptides and proteins in vivo (in preparation).

**Fig 8:**
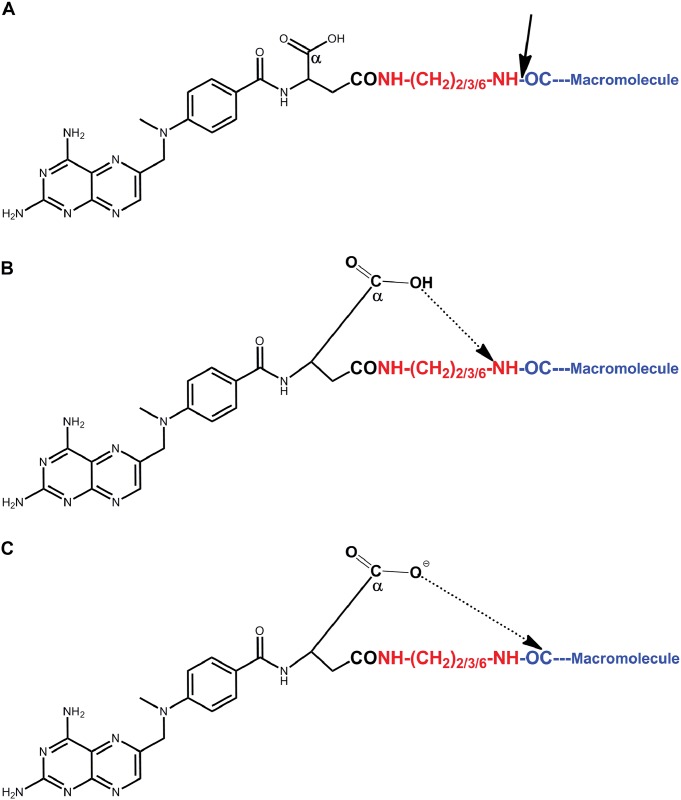
Schematic presentation of the proposed mechanisms involved in the chemical hydrolysis of methotrexate-amino derivatives covalently linked to carboxylate moieties of macromolecules (A) at acidic (B) and neutral (C) pH values.

We a priory assumed that the non-protonated α-carboxylate moiety of the MTX-amino derivative is essential to catalyze this peptide-bond cleavage. This carboxylate moiety has a pK_1_ of about 2.1 and it coincides well with the 5 fold accelerated rate of hydrolysis at pH 3.0 as opposed to pH 7.4 (Fig 4). Our preliminary attempts however to quantitatively derivatize the α side chain of MTX-CONH-(CH_2_)_6_-NH_2_ with glycinamide failed. This might be attributed to the low reactivity and/or steric hindrance of this moiety in the MTX-amino derivative. The finding that carboxylate linked Fmoc-glutamate-amino derivatives also undergo hydrolysis (Fig 5A and 5B), and the availability of Fmoc glutamic acid-amide, permitted us to demonstrate that carboxylate-linked Fmoc-glutamic-acid-amide-CO-γ-NH-(CH_2_)_6_-NH_2_ undergoes no hydrolysis (Fig 6C). The mechanism for this unprecedented hydrolysis is not clear. We propose however a possible mechanism which is brought in Fig 8. At both pH values, a state of pseudo ring structure is initially obtained. At acidic conditions hydrogen bond is formed between the α-carboxyl of glutamic acid and the amino group of the cleavable bond (Fig 8B) whereas at neutral pH, the α-carboxylate anion facilitates a nucleophilic attack on the carbonyl residue of the cleavable peptide bond (Fig 8C). Both pathways eventually lead to this amide-bond cleavage either at a faster (acidic) or a slower (neutral) fashion.

Interestingly enough, as observed here, all conjugates having an even number of ethylene groups (two or six) in the diamino spacer arm bridging MTX to the cleavable peptide bond (conjugates B, C, D, and E) hydrolyze at faster rates (t_½_ values of 6–17 min at pH 3.0, Table 3). The exception was conjugate F which has an uneven number (three ethylene groups) in the spacer connecting MTX to the cleavable peptide bond and hydrolyze at significantly lower rate (t_½_ = 58 ±3 min at pH 3.0, Table 3). This finding suggests that the pseudo ring structure formed in the case of conjugate F is considerably more stable, thus undergoing hydrolysis at a considerably lower rate. From practical stand point, the result implies that introducing MTX to albumin through a diamino spacer arm having an uneven number of ethylene groups is preferable if one wishes to obtain slower rates of hydrolysis.

Finally, we have studied in a MTX-sensitive CNS-1 glioma cell line, the antiproliferative efficacy of a cysteine specific BSA-MTX derivative, as opposed to the nonhydrolyzable (lysine-linked) BSA-MTX, and found that the former has 2.7 times higher antiproliferative efficacy and preserves nearly 50% the antiproliferative potency of the free, non-covalently linked MTX-hexamethylenamine (Fig 7, Table 4). These findings strongly suggest that this MTX-amino compound is released effectively from the conjugate, following internalization. It is of interest to determine if the released MTX-amino compound is ‘trapped’ inside the cell and/or the chemically cleavable bond is susceptible to intracellular enzymatic hydrolysis as well, two intracellular events that can explain this enhanced level of cytotoxicity (under study).

With regards to conjugate L, the amide bond between MTX and the ε-amino side chains of lysines was documented to be chemically and enzymatically stable [23]. We therefore hypothesize that the reactivation of such conjugates are likely to take place only following internalization into cells and massive lysosomal proteolysis.

In conclusion, we show here using cell-free as well as cell-based assays that carboxylate linked MTX-amino derivatives in particular, and carboxylate linked R-α-GLU-γ amino compounds in general are equipped with a ‘built-in chemical machinery’ that releases them under mild acidic conditions and suggest that this non-orthodox conjugation might have beneficial therapeutic advantages in the field of protein-drug conjugates.

## Abbreviations

BSA: bovine serum albumin
DCC: N-N’-dicyclohexylcarbodiimide
DHF: dihydrofolic acid
DHFR: dihydrofolate reductase
DIPEA: N,N’-diisopropylethylamine
DMF: dimethylformamide
DMSO: dimethylsulfoxide
DTNB: 5,5’-dithio-bis-(2 nitrobenzoic acid)
ESMS: electrospray single quandropole mass spectroscopy
GSH: reduced glutathione
HSA: human serum albumin
MAL: maleimide
MALDI-TOF: matrix-assisted laser desorption ionization time-of-flight
MEA: methotrexate-ethyleneamine (MTX-CONH-CH_2_-CH_2_-NH_2_)
MTX: methotrexate; PEG, polyethylene glycol
DCU: dicyclohexylurea
